# Climate Change Impacts on the Phenology of Laurentian Great Lakes Fishes

**DOI:** 10.1111/gcb.70436

**Published:** 2025-08-19

**Authors:** Morgan L. Piczak, Ava J. A. Sergio, Robert J. Lennox, Tys Theysmeyer, Jennfier E. Bowman, Jonathan D. Midwood, Steven J. Cooke

**Affiliations:** ^1^ Department of Biology Dalhousie University Halifax Nova Scotia Canada; ^2^ Ocean Tracking Network Dalhousie University Halifax Nova Scotia Canada; ^3^ Royal Botanical Gardens Burlington Ontario Canada; ^4^ Fisheries and Oceans Canada Great Lakes Laboratory for Fisheries and Aquatic Science Burlington Ontario Canada; ^5^ Department of Biology and Institute of Environmental and Interdisciplinary Science Carleton University Ottawa Ontario Canada

**Keywords:** coastal wetlands, conservation, fisheries management, global warming, non‐native species

## Abstract

Freshwater ecosystems around the world are increasingly impacted by climate change, yet there remains a lack of long‐term empirical data on how these changes are manifesting. In the Laurentian Great Lakes, a globally significant freshwater system, fish and their habitats are expected to be affected by warming water temperatures and increasing risks of species invasions. Despite these projections, relatively few studies have documented whether such shifts are already occurring. Our objective was to assess how climate change has influenced the community and migration phenology of native and non‐native fishes that use coastal wetlands in the Great Lakes. To do so, we analyzed local summer water temperatures and a 27‐year dataset (1997–2023) comprising arrivals of 16 fish species intercepted at the Cootes Paradise Marsh Fishway, a common carp ( *Cyprinus carpio* ) exclusion barrier at the western end of Lake Ontario. Over the study period, we found that mean summer water temperatures increased by over 1°C, consistent with broader global warming trends. Using non‐metric multidimensional scaling, we observed a unidirectional shift in fish community structure over time, rather than cyclical fluctuations or stabilization, indicating sustained ecological change. Analyses on phenology revealed that first, peak, and last arrival dates occurred earlier over time, while the duration of presence at the Fishway decreased for both native and non‐native species. These results provide evidence that climate change is already altering the community and phenology of fishes in Great Lakes wetlands. More broadly, our findings contribute to the growing body of literature showing that climate‐driven phenological shifts are reshaping freshwater ecosystems globally, underscoring the need for adaptive, climate‐informed conservation and management strategies.

## Graphical abstract

Long‐term monitoring at a Great Lakes wetland revealed that fish are arriving earlier and spending less time in the system, coinciding with more than a 1°C rise in summer water temperatures. Over 27 years, the fish community has also shifted in composition, providing evidence that global climate change is already reshaping freshwater ecosystems.

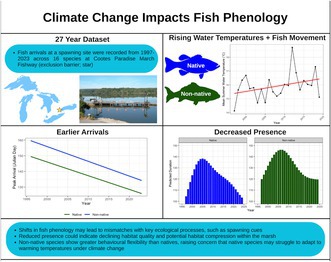

## 1 Introduction

Relative to other types of ecosystems (i.e., terrestrial and marine), freshwater ecosystems are experiencing biodiversity declines at much higher rates (WWF 2022) and are in a crisis state (Tickner et al. 2020). Since the 1970s, populations of freshwater species have decreased by more than 83% (WWF 2022) and one of the major threats stems from global climate change (Reid et al. 2019; Tickner et al. 2020). Specifically, climate change may jeopardize over 50% of freshwater fishes globally (Darwall and Freyhof 2016). Warming temperatures have the potential to impact species survival, distribution, and phenology, particularly in rivers and lakes where there is little scope for animals to shift their distributions northward, the way marine species are coping (Pörtner and Peck 2010). Despite many reviews on the topic exploring the potential impacts of climate change on freshwater fishes (Harrod et al. 2019; Ficke et al. 2007), there are still few examples of empirical changes in fish populations that can be attributed to climate change (Durance and Ormerod 2010; Myers et al. 2017; Hubbard et al. 2023, 2024; Koenigbauer et al. 2025).

Within the Laurentian Great Lakes of North America, coastal wetlands (i.e., those under substantial hydrologic influence from Great Lakes waters; McKee et al. 1992) provide critical habitat for fish, serving as spawning grounds, nurseries, and foraging areas (Jude and Pappas 1992). These wetlands, particularly littoral backwaters dominated by aquatic vegetation, are vital for many fish species that rely on vegetation for spawning (Jude and Pappas 1992; Midwood and Chow‐Fraser 2015; Trebitz and Hoffman 2015). Specifically, freshwater fish typically make seasonal movements into wetlands for foraging during the summer and exit the wetlands for overwintering in the lake (Schindler and Scheuerell 2002). Fish habitat in lakes Ontario, Erie, Huron, Michigan, and Superior is anticipated to be impacted by climate change through processes such as warmer water temperatures, reduced ice cover, prolonged stratification, and increased hypoxia (Collingsworth et al. 2017). These impacts are projected to interact with increased pressure from aquatic non‐native species that are predators, competitors, and parasites of native fishes in freshwater systems (Britton et al. 2023). In combination, alterations to freshwater habitats and impacts to biota from climate change and adverse effects associated with aquatic non‐native species are predicted to substantially impact the structure and function of fish populations and assemblages within the Great Lakes.

Despite predictions about the impact of climate change and biological invasions on aquatic ecosystems in the Great Lakes, there have been very few studies that have identified how these changes have manifested in a contemporary context (e.g., Wu et al. 2023; Hansen et al. 2017). Consequently, there is uncertainty about the trajectory of these freshwater ecosystems as warming accelerates (Collingsworth et al. 2017). Given that temperatures are projected to continue warming (Trumpickas et al. 2009), it will be important to advance our understanding of how climate change and invasions will impact the fish community of coastal wetlands. The objective of this paper was to examine the extent to which climate change is impacting the community composition and phenology of both native and non‐native fishes within the Great Lakes. To do so, we used a 27‐year dataset, consisting of temporal data for 16 fish species from a Lake Ontario wetland where they are monitored at the Cootes Paradise Marsh (CPM) Fishway that was installed to enable sorting of native and non‐native species (Chow‐Fraser 1998). This unique infrastructure provides data on the phenology of seasonal fish migrations over time (see Piczak, Theÿsmeÿer, et al. 2023; Boston et al. 2024). First, we examined changes in summer water temperatures using local temperature logger data to identify evidence of climate change locally. Next, we assessed how climate change may be impacting the phenology of Great Lakes fishes at three levels: community, species, and native versus non‐native status. We first examined changes in community composition within each year and across the full study period. We then assessed trends in first arrival for individual species and across native and non‐native groups. Finally, we evaluated trends in the duration of presence for native and non‐native species within Cootes Paradise Marsh. The aim of our study is to provide evidence to environmental managers regarding the impacts of global climate change on the fishes in coastal wetlands of the Great Lakes.

## 2 Methods

### 2.1 Study Site

**FIGURE 1:**
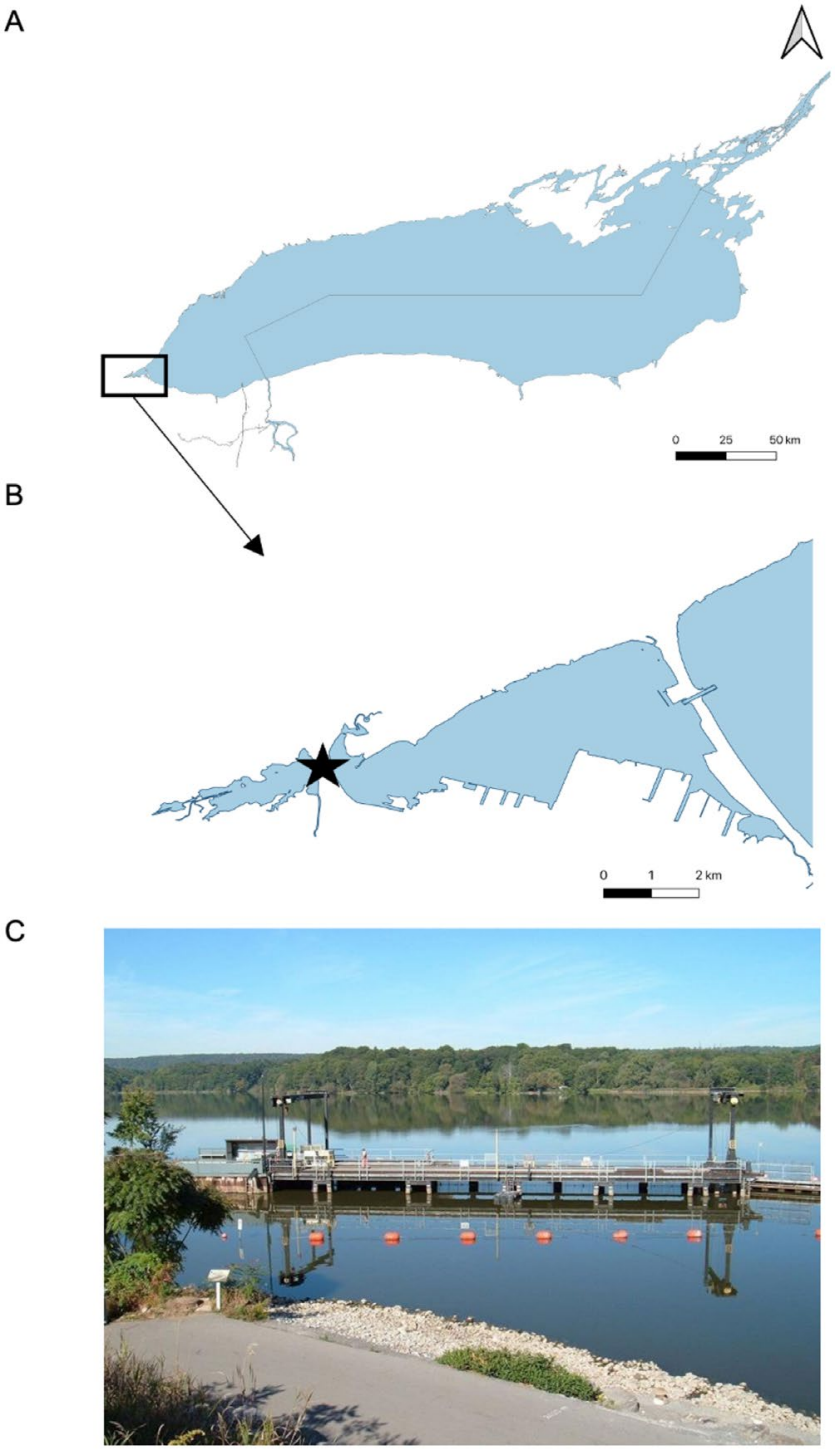
(A) Hamilton Harbour is located in the western end of Lake Ontario. (B) The Fishway (star) separates Cootes Paradise Marsh to the west, and Hamilton Harbour to the east. (C) A photo of the Fishway (photo credit: Royal Botanical Gardens).

Hamilton Harbour (HH), a 21‐km^2^ protected embayment, is located at the western end of Lake Ontario and is surrounded by the City of Hamilton (Figure 1). Situated at the western end of the harbour, CPM is a large (250 ha) degraded coastal wetland gradually recovering with dramatically changing habitat conditions as stressors including point source pollution are mitigated (Chow‐Fraser et al. 1998; Chow‐Fraser 2005; Theijsmeijer et al. 2023). Although CPM is located in a highly urbanized landscape, the marsh is regionally important owing to its high aquatic and terrestrial biodiversity (Chow‐Fraser 1998; Lougheed et al. 2004). Previous research has determined that the water temperature in Lake Ontario has increased by 1.6°C between 1968 and 2002 (Dobiesz and Lester 2009); however, we confirmed and examined trends locally. A chain of temperature loggers was deployed at the center of HH all year‐round (43.288131, −79.835487) and a summer temperature average was calculated for each year (June to August). These data were available until 2019; unfortunately, data was not collected after this point. While we did not have water temperature data for the last few years of our study, it is projected that air temperatures will continue to increase after 2019 via climate change (Channell et al. 2022).

### 2.2 Fishway

A physical‐exclusion barrier, herein called ‘the Fishway’ (operated by the Royal Botanical Gardens since 1997) was built between the CPM and HH and was designed to exclude larger non‐native common carp which were negatively impacting the wetland ecosystem (Chow‐Fraser 1998; Piczak, Bzonek, et al. 2023). Fish arriving at the Fishway are moving westward from HH into CPM. Water flow through the barrier is driven by both upstream watershed inputs through CPM and lake‐harbour seiches. The goals of the structure were to both prevent access of common carp large enough to physically damage marsh habitat (i.e., uprooting of macrophytes, muddying the water) and to reduce their spawning recruitment by eliminating females over 3 years old (HHRAP Fish and Wildlife Project Steering Committee 1993). The spacing of the vertical strainer bars is 5.0 cm, which is intended to permit passage of small fish; however, larger individuals of fishes are not able to pass through. The Fishway has cages, where all larger fish intending to migrate into the marsh become entrapped and are then manually sorted (i.e., selective passage); native species are released into the CPM, and common carp and other non‐native species (e.g., Goldfish, *Carassius auratus* ) are returned to HH (Theÿsmeÿer 1999). The Fishway has been successful at decreasing common carp biomasses by up to 95% in CPM (Theÿsmeÿer 1999; Lougheed et al. 2004) and has also helped reduce their biomass and abundance in HH (Boston et al. 2016).

### 2.3 Operation

Operation and passage of fishes at the Fishway initiates each year at ice‐off, typically between early March and early April, and continues until October when fall spawners have finished their migration. This condition has varied dramatically over the period of operation, with ice‐off dates trending earlier over time. During the winter period, the low typical seasonal lake level results in water depths of less than 0.5 m, with the Fishway not operated during the winter months. During operation, up to six cages are available for fish; four cages can be opened for inbound fish and up to two for outbound fish (i.e., leaving CPM to enter HH). During the spring, cages are typically lifted twice a day, Monday to Friday, to maintain pace with fish numbers, which typically occurs from the initiation of operation (i.e., ice‐off) to the end of June. As fish migration slows or concludes for many species, cage operations are reduced to once daily and eventually to three times per week during the limited summer migration period. While the species of every fish that enters the Fishway is recorded, a smaller subset of entrapped fish is measured (i.e., fork length in mm, mass in g) and sex noted when possible. Data from the Fishway were available from 1997 to 2023. Fish were either classified as native (i.e., bigmouth buffalo, *Ictiobus cyprinellus* ; gizzard shad, *Dorosoma cepedianum* ; bowfin, *Amia calva* ; brown bullhead, *Ameiurus nebulosus* ; channel catfish, *Ictalurus punctatus* ; freshwater drum, *Aplodinotus grunniens* ; largemouth bass, *Micropterus nigricans*; northern pike, *Esox lucius* ; white sucker, *Catostomus commersonii* , and yellow perch, *Perca flavescens* ) or non‐native (i.e., common carp, goldfish, rudd, *Scardinius erythrophthalmus* ; rainbow trout, *Oncorhynchus mykiss* ; white bass, *Morone chrysops* ; and white perch, *Morone americana* ). Small‐bodied fishes or life stages are able to move freely through the barrier, so are thus not captured in the Fishway monitoring program.

### 2.4 Statistical Analysis

#### 2.4.1 Community

All analyses were completed in R Statistical Environment Version 4.4.2 (R Core Team 2024). Ecological community at the Fishway was analysed using non‐metric multidimensional scaling (NMDS) with the vegan::metaMDS function with default Wisconsin double‐square root transformation (Oksanen et al. 2013). Species counts on each day of the Fishway operation were filtered to remove any rows with zero catches. Each row of the data represented a day of Fishway operation across years; therefore, each point in the model output estimated the community composition in two‐dimensional space within and across years. The period was divided into before or after July 1st, which lies about halfway between the median start and end dates in the time series, and the centroid of the polygon was calculated for each year using the car::dataEllipse function. The centroid of the ellipses was then plotted in ordinated space with segments connecting each year. A permutated analysis of variance was implemented using vegan::adonis2 to analyse the community trends as a function of day of the year, year, and water temperature within the dataset.

#### 2.4.2 Arrival, Peak and Last Arrival

Next, we investigated trends in the Julian dates annually for each species to characterize changes in phenology over time by calculating first, peak and last arrivals. We plotted each of these dates for each species across years with a linear trendline to visually examine temporal trends. To calculate peak arrivals, we converted daily counts into the cumulative proportion of the annual total for each species. We then identified the Julian date corresponding to 50% of the cumulative total for each species and year (following Piczak, Theÿsmeÿer, et al. 2023). We then used first, peak and last arrivals (in Julian dates) for each year per species as the response variable in three generalized additive models (GAM) using a Gaussian distribution using the ‘mgcv’ package with the mgcv::gam function (Wood 2011). The models each included an interaction between year and a categorical term for native versus non‐native species, with species included as a random effect. Each observation represented the first, peak, and last arrival for each year for each species. The smooth terms were defined with an appropriate number of basic functions to balance model flexibility with interpretability, reduce wiggliness, and prevent overfitting (as per Pedersen et al. 2019): 𝑘 = 5. Model performance was evaluated using residual plots from the gratia package (Simpson 2024).

#### 2.4.3 Duration

Temporal trends in duration (i.e., the maximum Julian date minus the minimum Julian date) for each species at the Fishway were modelled with a GAM using the mgcv::gam function with a negative binomial distribution. Duration was modelled as a function of an interaction between year and a categorical term for native versus non‐native species. Species was included as a random effect. We included smooth terms (*k* = 5), with a thin plate spline. Most counts were small (IQR = 1–8), so extreme values > 100 were removed as they disrupted the residuals. Model performance was assessed using the same methods as the GAM for arrivals.

## 3 Results

### 3.1 Water Temperature

**FIGURE 2:**
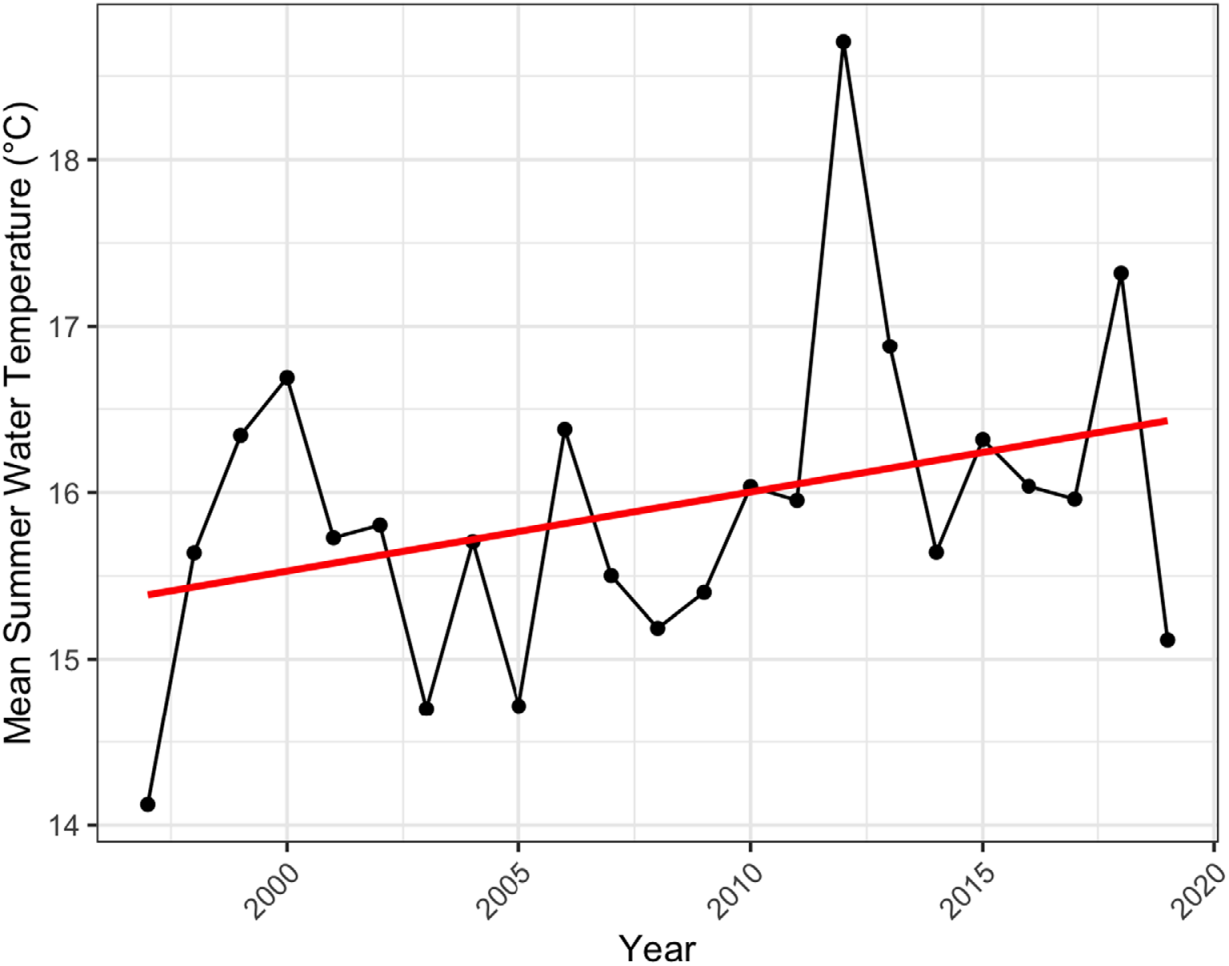
Increase in mean summer water temperatures with a linear trendline (red) from Hamilton Harbour throughout the study period.

Mean summer water temperatures increased throughout the study period, although there was a high degree of interannual variation (Figure 2). Specifically, the average summer temperature increased by over 1.0°C, from 15.4°C in the mid 1990's to 16.4°C in 2019 (Figure 2).

### 3.2 Community

**FIGURE 3:**
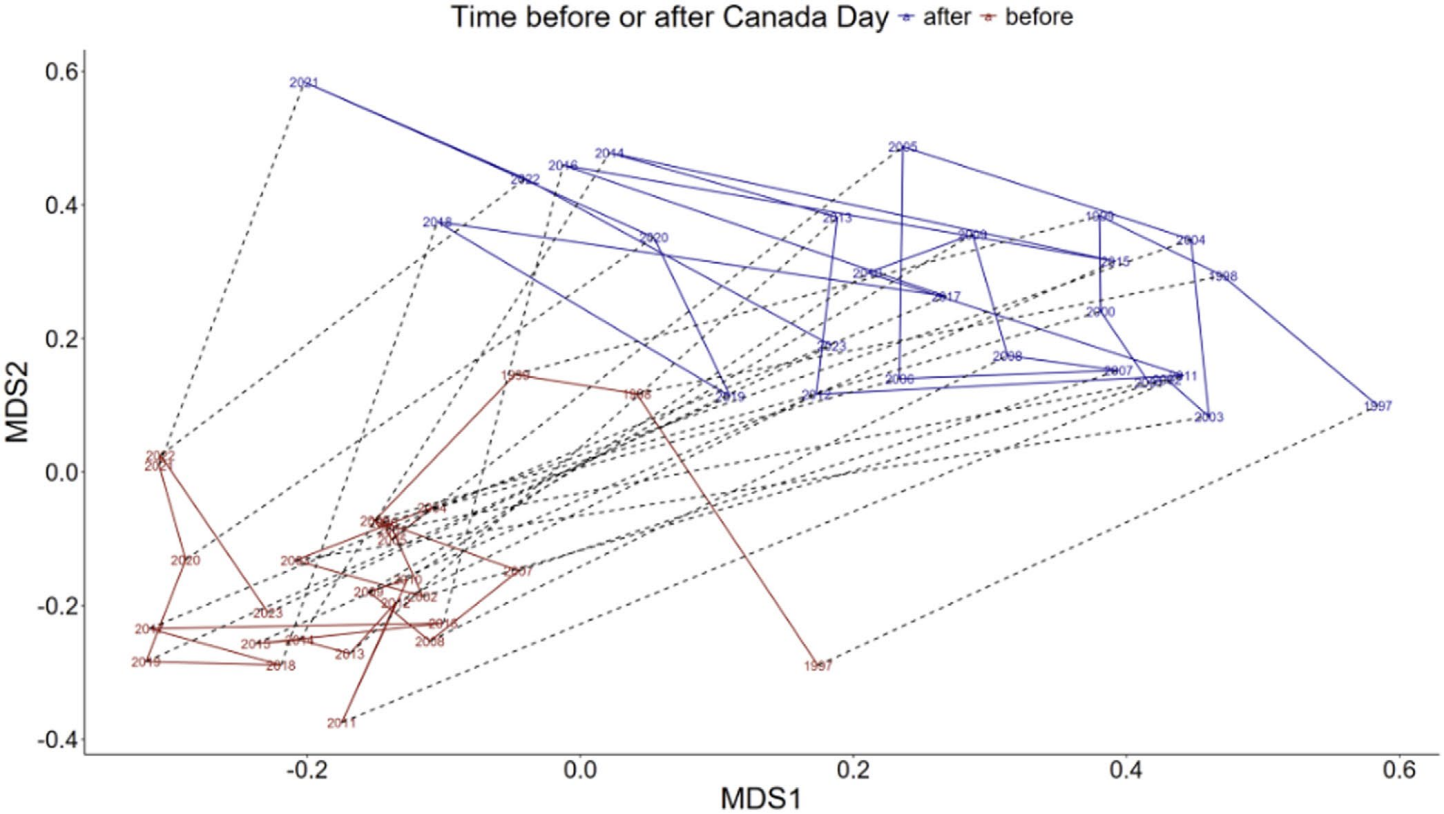
Centroids of the annual NMDS results by year and period (before or after July 1 of sampling year). Periods within years are connected to each other by dashed lines. Both periods demonstrate a gradual temporal shift in the community structure from left to right across time.

The fish community at the Fishway has undergone a gradual shift across the 27‐year time series, which was paralleled by the increase in water temperatures during the same time period (Figure 3). The NMDS analysis had a stress value of 0.24, slightly higher than what is considered to be ideal for a good fit. We did not attempt to subset the data to achieve a lower stress value. The NMDS showed that fish communities in both the first and second halves of the year have followed a consistent directional shift across time towards a different species composition. Permutated analysis of variance identified strong and significant changes within years (*F* = 637, *p* < 0.01), across years (*F* = 174, *p* < 0.01), and with temperature (*F* = 51, *p* < 0.01). There was a greater spread in the community composition after July 1st compared to the first half of the year, suggesting increased variability in the species composition later in the season. While the absolute direction in ordination space is arbitrary due to scale invariance, both seasonal communities have progressively moved across the ordinated space, indicating a long‐term shift in community structure that has (so far) not returned towards the baseline and may continue to change in future years along the same trajectory. If the community structure were stable, we would expect the time series to be stationary; if community composition were cyclical, we would expect the years to bounce back and forth from one side to the other; if composition reached a stable point, we would expect the lines to move and then stop, with years overlapping once the composition stabilised. Instead, the separation of years over time indicates ongoing ecological change. While there is some stability within each year, changes between years were more pronounced, reinforcing the interpretation that the fish community is experiencing directional change rather than annual fluctuations that return to an equilibrium state.

### 3.3 Arrival, Peak, and Last Arrival

**FIGURE 4:**
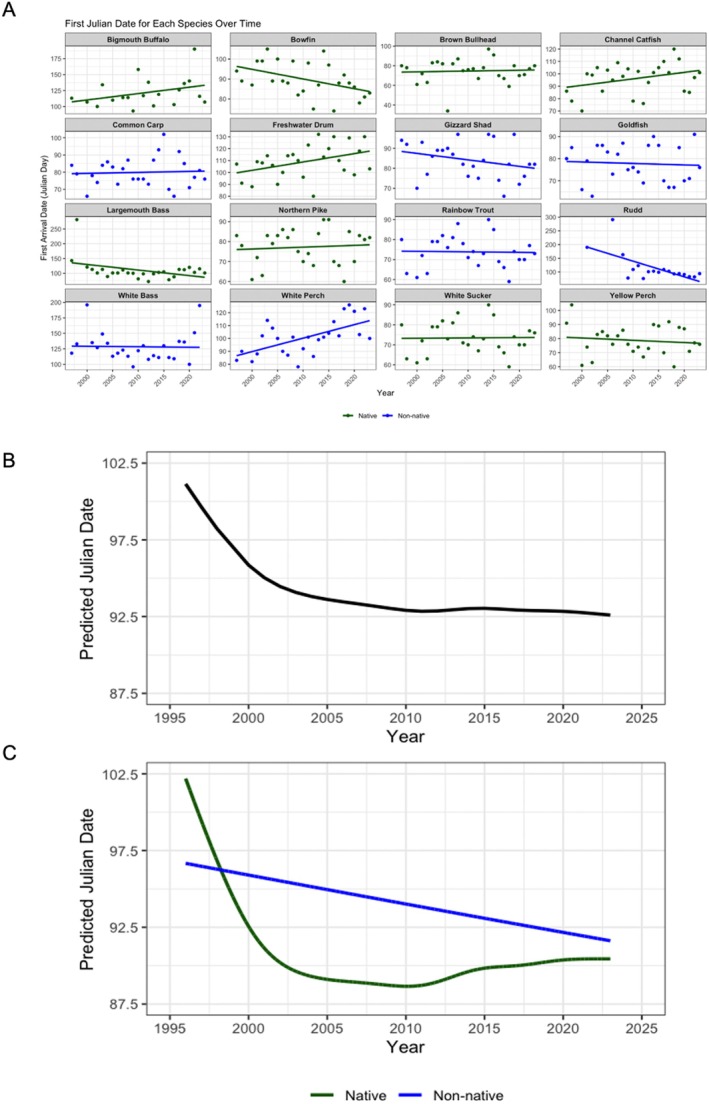
(A) First arrival (at the Fishway in Julian days) by species per year over time for both native and non‐native fishes, with trendlines. (B) The predicted first arrival (in Julian days) for all fishes in the Laurentian Great Lakes, and (C) split into native and non‐native fishes over time.

Next, we investigated trends in the first arrival, peak movements, and last arrival at the Fishway in Julian day over time. First, we found that typically the majority of species were arriving earlier year over year, consistent with the rising water temperatures (Figure 4a). For example, both northern pike and yellow perch had a shift toward earlier arrivals throughout the study period (Figure 4a). There were a few exceptions; for instance, bigmouth buffalo and freshwater drum (both native) and white perch (non‐native) arrived later toward the last few years of the study (Figure 4a). The additive model of first arrival found no differences between native and non‐native species (SE = 10.0 and *p* = 0.81; Figure 4b; see Data S1 for estimated smooth effects). Across both native and non‐native species, the GAM predicted earlier arrivals over the years, in that for 1997, fish were predicted to arrive at approximately day 101, which decreased and plateaued to 92 by 2023, representing a change in arrivals of over a week earlier (Figure 4b). Although the interaction between native fish and year was not significant (*F* = 1.79 and *p* = 0.09), there was a sharp decrease in the arrival time for native species between 1997 and 2005, where the predicted first arrival decreased by almost 15 days (Figure 4c). After 2005, the additive model predicted a plateau in arrivals for native species, where the predicted arrival hovered around 88 (Figure 4b). Overall, the predicted first Julian day of native fishes was earlier over time by over 10 days across the study period (Figure 4c). The interaction between the arrival of non‐native species and year was significant (*F* = 3.33 and *p* = 0.04), whereby the predicted Julian day was earlier across years, which was in alignment with the warming water temperatures (Figure 4c). In the earlier years, non‐native species were predicted to arrive after Julian day 96, and over the 25‐year period, it decreased to Julian date 92 (Figure 4c). Relative to the native fishes, the non‐native species were predicted to arrive less early over time, by approximately 5 days (Figure 4c).

**FIGURE 5:**
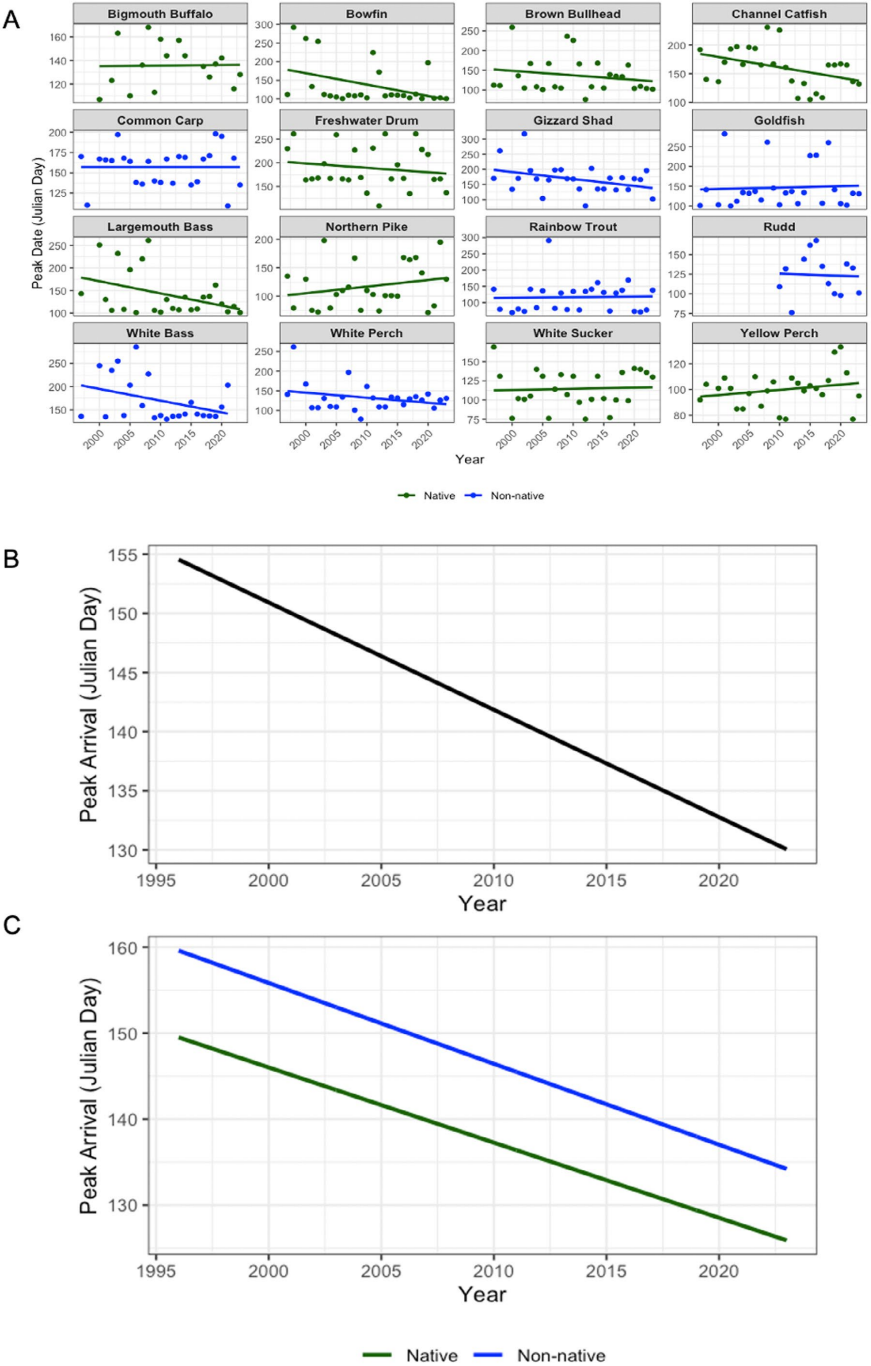
(A) Peak arrival (when cumulatively 50% of each species arrived at the Fishway in Julian days) by species per year over time for both native and non‐native fishes, with trendlines. (B) The predicted first peak arrival (in Julian days) for all fishes, and (C) split into native and non‐native fishes over time.

Next, we investigated trends in peak movement timing at the Fishway in Julian day over time. Similar to first arrival, we found that most species tended to reach their peak movement earlier across the years, likely in response to rising water temperatures (Figure 5a). For instance, bowfin, channel catfish, and largemouth bass (all native) showed clear shifts toward earlier peak movements. In contrast, a few species, including northern pike and white sucker (native), as well as goldfish (non‐native), showed relatively stable or slightly delayed peak movements across the study period (Figure 5a). The GAM found no significant difference between native and non‐native species overall (Estimate = 9.15, SE = 11.81, *p* = 0.43; Figure 5b; see Data S2 for estimated smooth effects). The GAM analysis revealed that native species showed significantly earlier peak movement dates over time (*F* = 6.49, *p* = 0.01; Figure 5c), with predicted peak dates shifting from approximately Julian day 148 in 1997 to 127 by 2023. Non‐native species also showed a significant trend toward earlier peak movements (*F* = 5.24, *p* = 0.023; Figure 5c), with the model predicting a decrease from day 159 in 1997 to about 140 by 2023.

**FIGURE 6:**
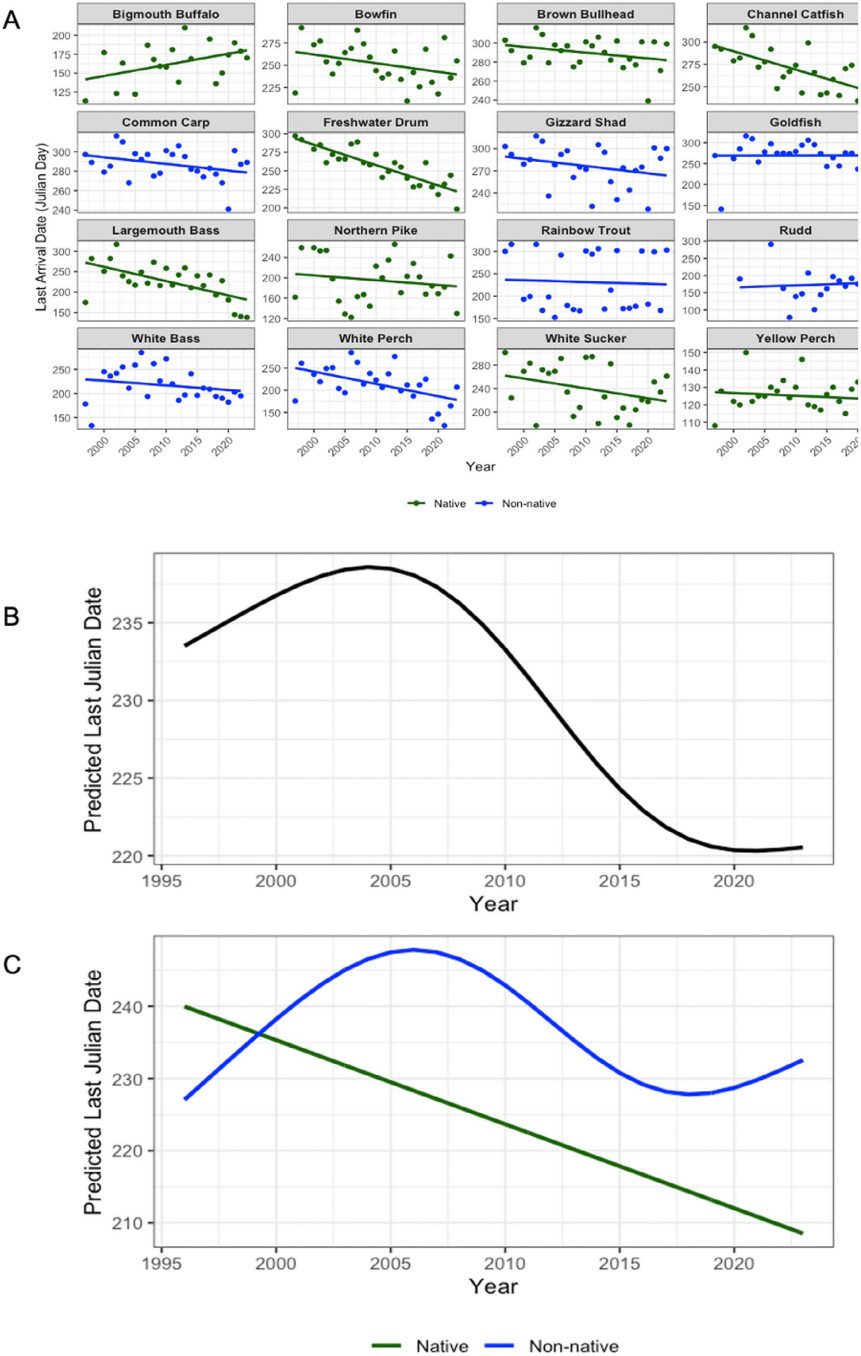
(A) Last arrival (at the Fishway in Julian days) by species per year over time for both native and non‐native fishes, with trendlines. (B) The predicted last arrival (in Julian days) for fishes in the Laurentian Great Lakes, and (C) split into native and non‐native fishes over time.

Finally, we investigated trends in last arrival timing at the Fishway in Julian day over time. Similar to patterns observed for first arrival and peak movement, many species showed earlier last arrival dates over the study period (Figure 6a). For example, channel catfish, largemouth bass, and freshwater drum (all native) demonstrated earlier last arrivals across the study period, while species such as bigmouth buffalo and rudd showed relatively later last arrivals over time (Figure 6a). Among non‐native species, most (e.g., white perch, gizzard shad and common carp) also showed modest declines in last arrival dates, although others like goldfish and rainbow trout remained relatively stable across years (Figure 6a). The GAM found no significant difference in overall last arrival timing between native and non‐native species (Estimate = 9.15, SE = 11.81, *p* = 0.439; Figure 6b; see Data S3 for estimated smooth effects). However, both groups demonstrated significant temporal trends. Native species showed a consistent decline in last arrival dates over the study period (*F* = 17.09, *p* < 0.001; Figure 6c), with predicted dates shifting from approximately Julian day 240 in 1997 to about 210 by 2023. In contrast, non‐native species exhibited a nonlinear trend (edf = 3.02, *F* = 2.46, *p* = 0.041; Figure 6c), with predicted last arrival dates increasing in the early 2000s, peaking around 2005, and subsequently decreasing before slightly increasing again toward 2023.

### 3.4 Duration

**FIGURE 7:**
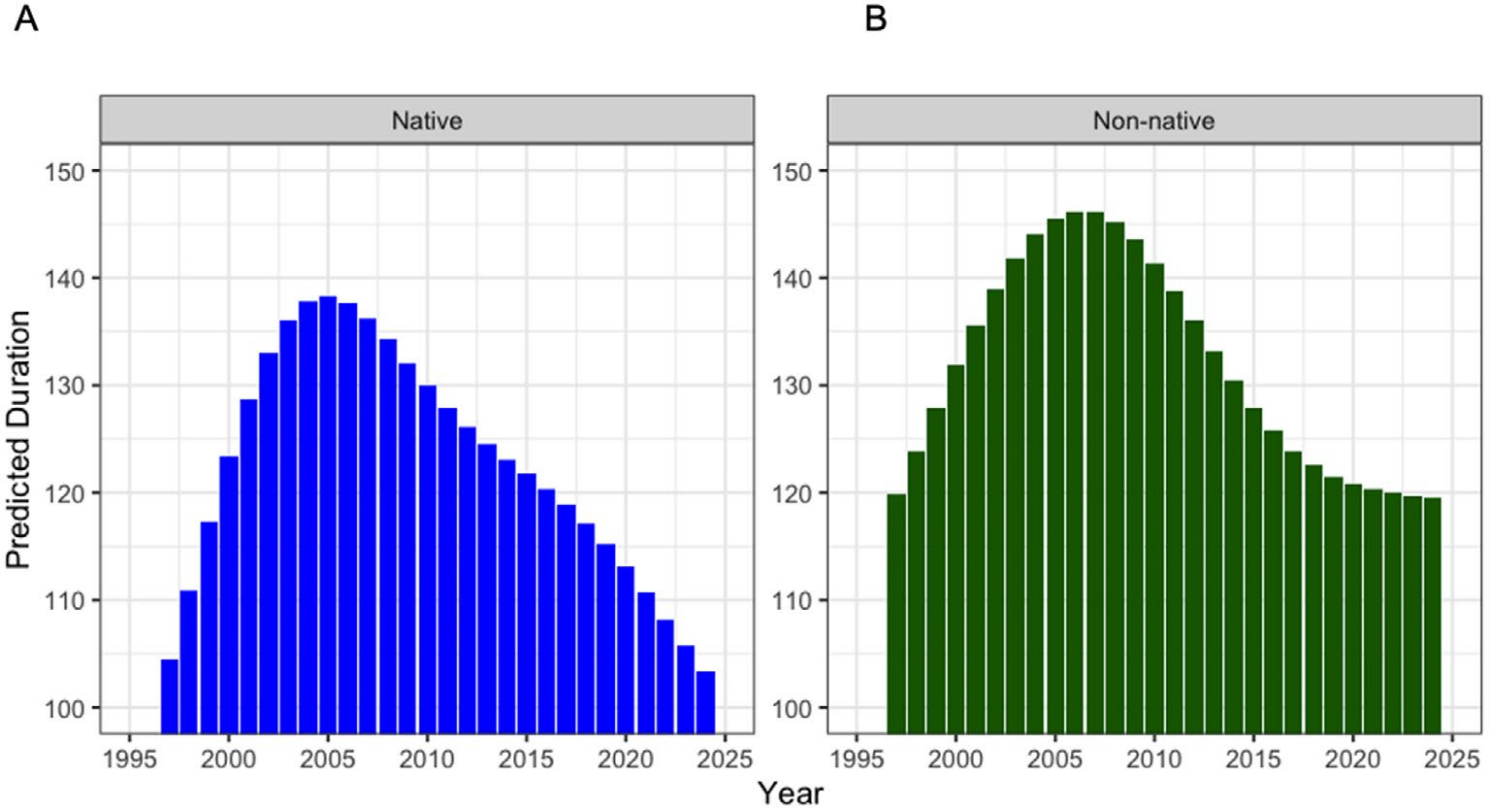
Additive model of duration (in days) for (A) native and (B) non‐native fishes at the Cootes Paradise Marsh Fishway.

The GAM of species counts throughout the year identified several important trends (Figure 6). The model revealed that year had a significant effect on duration for both native and non‐native species (*z* = 32.1 and *p* < 0.001; see Data S4 for estimated smooth effects). While there was no difference in duration across native and non‐native species (*z* = 0.296 and *p* = 0.75), there was a difference in how the duration of each group responded over time. Specifically, the interaction between year and native (*χ* ^2^ = 17.8 and *p* < 0.001) and year and non‐native were both significant (*χ* ^2^ = 8.65 and *p* < 0.04). At the start of the study period, the predicted duration for native species was shorter compared to non‐native species: approximately 105 and 120 days, respectively (Figure 7). The predicted duration of presence increased steadily for both native and non‐native species, peaking between 1995 and 2010, where there was an increase in duration by ~30 and 25 days for native and non‐native fish, respectively. Native species exhibited a sharper increase followed by a more pronounced and consistent decline after 2010, reaching levels similar to or lower than those observed in the late 1990s by 2025 (Figure 7a). In contrast, non‐native species maintained higher predicted durations for a longer period post‐peak, with a more gradual decline that began around 2010 and plateaued after 2020 (Figure 7b).

## 4 Discussion

We examined the impact of climate change on the community composition and phenology of native and non‐native fishes within the Great Lakes wetlands over 27 years. Using local loggers, we also determined that during the study period, the mean summer water temperature increased by over 1°C, which was paralleled by changes in fish community and phenology. Rather than experiencing annual fluctuations that return to a baseline, the fish community exhibited continuous long‐term directional change. The nature and direction of change in fish community composition indicated that CPM is experiencing ongoing ecological change across years, consistent with rising temperatures. The overall findings regarding phenology for each species were similar across both native and non‐native fishes. Both native and non‐native fishes exhibited earlier first, peak, and last arrival dates, along with a decline in the duration of presence within CPM over time. Here, we will discuss temporal trends in community and phenology, while also disentangling local versus climate‐driven patterns of change at the Fishway within CPM. Based on the identified trends and drivers of change, we will outline global management implications for fishes responding to climate change.

Our results indicate a consistent, directional shift in fish communities both across years and in the first and second half of the year towards different species composition. These results are consistent with a 30‐year study of the fish community structure in HH, located adjacent to the Fishway, which also found high inter‐annual variation in fish community and documented considerable shifts in community composition during the study period (Maynard et al. 2022). While trajectories of community change can be non‐directional or cyclical, returning to a previous baseline, our findings show a persistent shift across time, reinforcing the idea that the community is not stabilizing or reverting to earlier compositions (Matthews et al. 2013). In addition to climate change, declines in multiple native fish populations leading to reduced species richness throughout the HH area are likely also contributing to the observed changes in species composition (Midwood et al. 2024). Several basin‐scale changes in HH could be leading to reduced species richness and altering fish communities in the marsh, as other areas of Lake Ontario, such as the Bay of Quinte, have not experienced such drastic shifts in community (Turner et al. 2025). The re‐establishment of predatory walleye (OMNRF 2020), the novel establishment of non‐native species (e.g., rudd; Balshine et al. 2005; Boston 2013 unpublished data), and the significant decline in abundance of non‐native common carp could all be contributing locally to the long‐term community change (Boston et al. 2016). Still, the literature more strongly suggests that climate change may be a primary driver of these community shifts, operating in conjunction with system‐specific factors. Globally, changes in weather regimes (e.g., increased temperatures, low precipitation) caused by climate change have been found to negatively impact the abundance of native fish species while non‐native species prospered, resulting in significant changes in the structure of fish populations over time (Ilarri et al. 2022). Nonetheless, literature on this topic remains comparatively limited globally relative to marine ecosystems (e.g., Cheung et al. 2012; MacKenzie et al. 2007; Rijnsdorp et al. 2009) and the well‐documented phenological responses to climate change in terrestrial taxa, such as migratory birds (Miller‐Rushing et al. 2008; Visser et al. 2009; Zaifman et al. 2017). Much of what does exist for freshwater fishes consists of projections or modeled scenarios, rather than documented, in situ ecological change (Balcombe et al. 2011; Morrongiello et al. 2011; Macusi et al. 2015; Xenopoulos et al. 2005). To continue to disentangle how climate change is reshaping fish communities, long‐term empirical studies in freshwater environments would be beneficial.

To assess how the phenology of native and non‐native species has changed over time, we analyzed the timing of first, peak, and last arrivals at the Fishway, along with the duration of their migratory periods. Overall, we found that the migratory phenology of both native and non‐native fishes occurred earlier over time. These findings align with previous research that also documented changes to phenology in response to warming water temperatures within the Great Lakes (e.g., Lyons et al. 2015; Farmer et al. 2015). While many marine fish species have exhibited the potential to expand poleward in response to warming (Hastings et al. 2020), landlocked freshwater species are required to shift their range across complex freshwater systems or adapt in other ways, such as modifying seasonal movements. As climate change continues to drive water temperatures to increase, fish could seek to satisfy their need for cooler water temperatures by arriving earlier in the season (see Collingsworth et al. 2017; Comte et al. 2013). While the overall trend of earlier migrations could have unified consequences, the timing of first, peak, and last arrivals is likely governed by different ecological pressures. First arrival may reflect suitable upstream environmental conditions whereas peak arrival may be influenced by favorable conditions at the Fishway, such as water temperature and discharge rates. Native and non‐native species trends diverged significantly with the timing of last arrivals, where native species continued to depart earlier and non‐native species displayed a non‐linear trend over time. We found similar results between both groups from our duration analysis. After an initial peak in duration, native fishes exhibited a continual decline in duration for the remainder of the study period (form 2005 onwards) whereas the response of non‐native fishes was non‐linear and eventually plateaued. This initial peak in duration for both groups is believed to be the result of system‐specific changes at that time, such as habitat improvement due to mitigated invasive common carp and comparatively small increases in water temperature (Lougheed et al. 2004). Such shifts in arrivals and duration raise the possibility of mismatches between fish migratory timing and key environmental cues or trophic interactions, such as prey availability or optimal spawning conditions (Volkoff et al. 2010; Forsythe et al. 2012). These mismatches may contribute to phenological compression, a phenomenon often driven by climate change where the duration of specific life stages in organisms is reduced due to phenological shifts (Cappello and Boersma 2021; Tomotani et al. 2018).

Our findings suggest that shifts in phenology and the resulting compression of the migratory period underscore the critical role of behavioral flexibility in enabling fish to respond to environmental change (Beever et al. 2017). Through earlier arrivals at the Fishway and shorter durations, both native and non‐native species exhibited behavioural flexibility in response to climate‐driven environmental changes across time; however, the non‐linear response of invasive species for both last arrival and duration suggests that they may have a greater capacity for change. Non‐native fishes can be more tolerant of environmental changes due to their broader physiological range, opportunistic life histories, and plasticity in habitat use (Beever et al. 2017), therefore explaining the lesser response in last arrivals and duration compared to native fishes. Not only do these traits often enable non‐native fishes to outcompete native species in changing ecosystems, but they could also allow for a tradeoff between the degree of behavioural flexibility and the ability to better tolerate stressors. Non‐native species, such as goldfish, have exhibited the ability to withstand traditionally unfavourable conditions such as lower dissolved oxygen and high water temperatures (Sollid et al. 2005; Richardson et al. 1995). Non‐native species may also be able to better tolerate environmental disturbance and anthropogenic stressors (Boston et al. 2016; Trebitz et al. 2009). This is particularly relevant in CPM, where a long history of anthropogenic degradation from point and non‐point source pollution, vegetation loss, high turbidity, and altered food webs has created a challenging living environment (Chow‐Fraser 1998). Globally, behavioural flexibility has been well documented in freshwater, coastal, and tropical fish species in response to climate change through alterations to spawning, feeding, and swimming (Heatwole and Fulton 2012; Rodriguez‐Dominguez et al. 2019; Hovel et al. 2017). As such, our findings have broader implications for freshwater wetland systems worldwide, many of which are experiencing similar shifts in temperature regimes and hydrological patterns due to climate change.

Globally, freshwater wetlands are among the ecosystems most severely threatened by climate change (Mitsch and Hernandez 2013; Junk et al. 2013; Lynch et al. 2023). Like CPM, many freshwater wetlands tend to be shallow and eutrophic (Kim et al. 2016), making them susceptible to exceedingly warm water temperatures and associated hypoxia. In wetlands where hydrological cycles are tightly linked to seasonal precipitation or snowmelt (Bullock and Acreman 2003), earlier or compressed migration windows could disrupt critical ecological interactions, such as synchrony with food resources, spawning substrates, or wetland inundation timing (Andersen et al. 2000; Turner and Boesch 1988). The potential for phenological compression, particularly for native species, raises concerns that species lacking sufficient behavioral plasticity may be at heightened risk of phenological mismatches, causing trophic displacement or reproductive failure (Both et al. 2009; Walker et al. 2019). This phenomenon is also relevant in tropical and subtropical wetlands, where many species are already living near their thermal thresholds and may face limited options for spatial movement or timing shifts in search of thermal refugia (Gopal 2013; Waltham and Schaffer 2018). Moreover, the apparent capacity of non‐native species to adjust their migratory timing in more variable or non‐linear ways suggests that these species may have a competitive advantage in other wetland systems undergoing rapid environmental change. Non‐native fishes with broad thermal and ecological tolerances could increasingly dominate wetland communities, particularly in regions where native species have narrower climatic niches or more rigid life history strategies, such as temperate floodplain wetlands, subtropical deltas, or tropical swamp forests (Abbey‐Lee et al. 2013; Costa‐Pereira et al. 2017). In these areas, similar patterns of phenological shifts and invasion dynamics may already be occurring, yet remain under‐documented due to a lack of long‐term, fine‐scale data in many of these systems. Comparative research across biogeographic regions is needed to assess the generality of these patterns and to inform management strategies aimed at conserving vulnerable native species under shifting climate regimes. Specifically, future studies that directly incorporate key environmental covariates (e.g., water temperature, flow) would be beneficial to clarify the mechanistic drivers of the observed trends.

## 5 Global Management Implications

Our findings highlight three key management priorities that are broadly applicable to freshwater systems facing climate change: the need for climate‐adapted operation of exclusion barriers, the implementation of climate‐resilient ecological restoration, and the critical importance of sustained, high‐resolution monitoring to track ongoing ecological shifts. According to a global review, many active exclusion barriers are not operated based on the phenology of fishes (Piczak, Bzonek, et al. 2023), despite evidence that fish migration timing is shifting inter‐annually in response to climate change. Our results show that both native and non‐native fishes are adjusting their arrival timing with warming temperatures, yet many exclusion structures continue to function on fixed schedules. In contrast, the Fishway is managed more flexibly, allowing adjustments based on observed fish arrival and local weather conditions. Given our finding that non‐native species are arriving earlier each year, we recommend that exclusion barriers currently operating on rigid schedules be adapted to align with these phenological shifts. Doing so will help maintain the effectiveness of managing non‐native species under changing climate regimes. Moreover, there is growing evidence that seasonal operation of exclusion barriers can also support native species (Piczak, Theÿsmeÿer, et al. 2023). This is particularly important in systems with highly migratory species, where fixed operating periods may disrupt seasonal movements and prevent upstream passage. Mismatches between barrier operation and the evolved migratory timing of fish can have serious consequences, including reproductive isolation when species using the same spawning habitats arrive at different times (Quinn et al. 2000; Tamario et al. 2019). Proactively adapting exclusion barrier operations to account for shifting phenology and increasing climate variability is critical to ensure the long‐term success of invasive species management while supporting native fish populations.

Given the decreasing duration of native fishes within CPM over time, we recommend that ecological restoration be guided by a future‐proofing lens (Piczak, Perry, et al. 2023; Lynch et al. 2023). Locally, the historic loss of aquatic vegetation in the marsh has led to the collapse of creek channels that once served as critical stormwater reservoirs (Bowman, personal comm.). Additionally, ongoing watershed erosion and infilling have altered sediment dynamics, resulting in increased suspended sediment concentrations. Restoration efforts aimed at reducing sediment accumulation in creeks and marshes would benefit cold‐water, native‐dominated ecosystems by expanding suitable habitat, promoting aquatic vegetation regrowth, and supporting insect abundance and diversity. More broadly, these restoration strategies could serve as a model for other systems experiencing similar challenges. Habitat degradation and warming temperatures are reducing the duration of fish presence in many freshwater ecosystems worldwide. Restoring habitat quality is therefore a key step toward enhancing phenological stability and ecosystem resilience. As climate change progresses, the availability of thermal refugia is becoming increasingly important for sustaining aquatic species (Railsback and Harvey 2023). Yet, thermal suitability in freshwater habitats is declining globally (Wenger et al. 2011; Isaak and Rieman 2013; Svenning et al. 2022). Restoration that maintains or enhances thermal refugia can help buffer species against these stressors and has been shown to increase the resilience of native species to climate change (Arriaga et al. 2024). Despite the growing recognition of these benefits, examples of climate‐resilient restoration remain limited (Simonson et al. 2021). Adopting a future‐proofing approach is essential to ensure long‐term restoration success (Frietsch et al. 2023) and has proven effective in enhancing adaptive capacity where implemented (Simonson et al. 2021; Lynch et al. 2023). As such, restoration efforts should prioritize areas with high recovery potential—both to strengthen the resilience of ecosystems and to proactively address future environmental stressors. The case of CPM offers a locally grounded but globally relevant example of how restoration can be adapted to meet emerging ecological challenges under climate change.

This study benefited from an exceptionally high‐resolution dataset, with daily sampling that enabled the detection of long‐term temporal trends in fish community dynamics. Such fine‐scale temporal resolution allowed us to observe realized ecological changes over time, rather than relying solely on predicted or modeled effects of climate change. Through sustained monitoring, our study underscores the global importance of high‐frequency sampling over extended periods to understand ecological changes that unfold across decades. Despite these benefits, continuous high‐resolution monitoring remains rare at the global scale. There are well‐documented deficits in long‐term freshwater biodiversity data in regions of critical importance, including the Amazon Basin (Castello et al. 2013), parts of Europe (Lepetz et al. 2009), and many other biodiverse but data‐poor areas. Although such intensive sampling programs are not always feasible, their value is substantial. High‐frequency, long‐term data improve the accuracy and reliability of ecological and climate models, enhance our ability to detect shifts in species composition or phenology, and reduce the risk of overgeneralization that can occur with coarse or sporadic datasets (Lepetz et al. 2009). Wherever possible, we recommend that similar high‐resolution, long‐term monitoring approaches be adopted globally. These efforts are essential for equipping resource managers and policymakers with the actionable, site‐specific information required to respond to rapid ecological change. As climate‐driven impacts accelerate, such monitoring programs are critical for detecting early warning signs, informing adaptive management, and ultimately preserving the resilience of freshwater ecosystems worldwide.

## 6 Conclusion

Freshwater ecosystems across the globe are facing mounting pressures from climate change, habitat degradation, and species invasions, drivers that are fundamentally altering biodiversity and ecosystem function. Our study provides robust, long‐term empirical evidence that climate change is reshaping the fish commiunity and phenology in coastal wetlands of the Laurentian Great Lakes. Over a 27‐year period, we documented a consistent, unidirectional shift in fish community structure, alongside earlier seasonal arrivals, peaks, and last arrivals, as well as a shortened duration of presence for both native and non‐native species. These findings reflect a broader global trend: species with broader environmental tolerances are increasingly favored under climate stress, while native biodiversity faces heightened risk. Although rooted in a North American context, our findings have global relevance. Wetlands worldwide are experiencing parallel threats, including habitat loss, warming waters, and impacts associated with non‐native species. To support freshwater biodiversity in the face of accelerating climate impacts, we identify three globally applicable management priorities: (1) adaptively managing exclusion barriers and fish passage infrastructure in response to changing phenological patterns; (2) designing ecological restoration projects that enhance climate resilience, particularly through the protection and restoration of thermal refugia; and (3) establishing and maintaining high‐frequency, long‐term monitoring programs to guide adaptive responses. As freshwater ecosystems globally continue to warm and degrade, these approaches will be essential to safeguard biodiversity, ecosystem services, and the cultural and economic values tied to freshwater systems. The case of changing community composition and fish phenology within CPM offers a globally relevant example of how local, long‐term ecological insight can inform the adaptive, climate‐resilient management urgently needed across freshwater ecosystems worldwide.

## Author Contributions

**Morgan L. Piczak:** conceptualization, data curation, formal analysis, investigation, methodology, project administration, resources, supervision, validation, visualization, writing – original draft, writing – review and editing. **Ava J. A. Sergio:** writing – original draft, writing – review and editing. **Robert J. Lennox:** conceptualization, formal analysis, investigation, methodology, project administration, supervision, validation, visualization, writing – original draft, writing – review and editing. **Tys Theysmeyer:** data curation, funding acquisition, methodology, project administration, supervision, writing – review and editing. **Jennfier E. Bowman:** data curation, funding acquisition, project administration, writing – review and editing. **Jonathan D. Midwood:** conceptualization, project administration, supervision, writing – review and editing. **Steven J. Cooke:** project administration, resources, supervision, writing – review and editing.

## Conflicts of Interest

The authors declare no conflicts of interest.

## Supporting information


**Data S1:** gcb70436‐sup‐0001‐DataS1.zip.

## Data Availability

The data and code that support the findings of this study are openly available in Zenodo at https://doi.org/10.5281/zenodo.16322241 and https://doi.org/10.5281/zenodo.16618918, respectively.
