# Supplementary material for: Climate Change Impacts on the Phenology of Laurentian Great Lakes Fishes
Source: Glob Chang Biol. 2025 Aug 19;31(8):e70436. doi: 10.1111/gcb.70436 (PMC12365581; doi:10.1111/gcb.70436)
Supplement: Supplementary file 1 — Data S1: gcb70436‐sup‐0001‐DataS1.zip. [file GCB-31-e70436-s001.zip › Supinfo/GCB_SI.docx]

# **Supplemental Information**


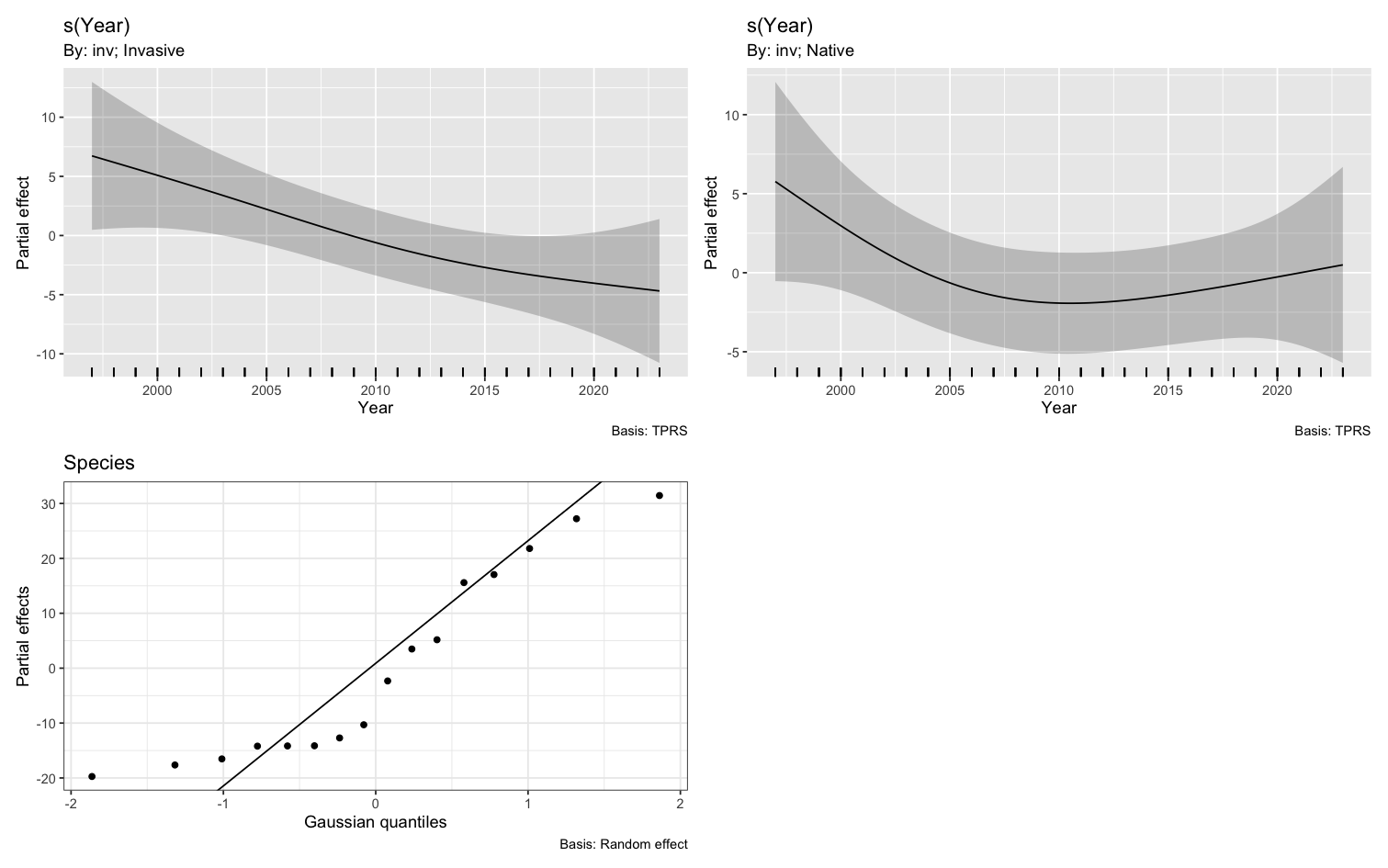


Supplemental 1. Estimated smooth effects from the generalized additive model showing temporal trends for the first arrival for native and non-native fish species. Top panels display the smooth effect of year on last occurrence by invasion status (Native, Non-native), with shaded areas representing 95% confidence intervals, and bottom shows the random effect estimates for individual species.


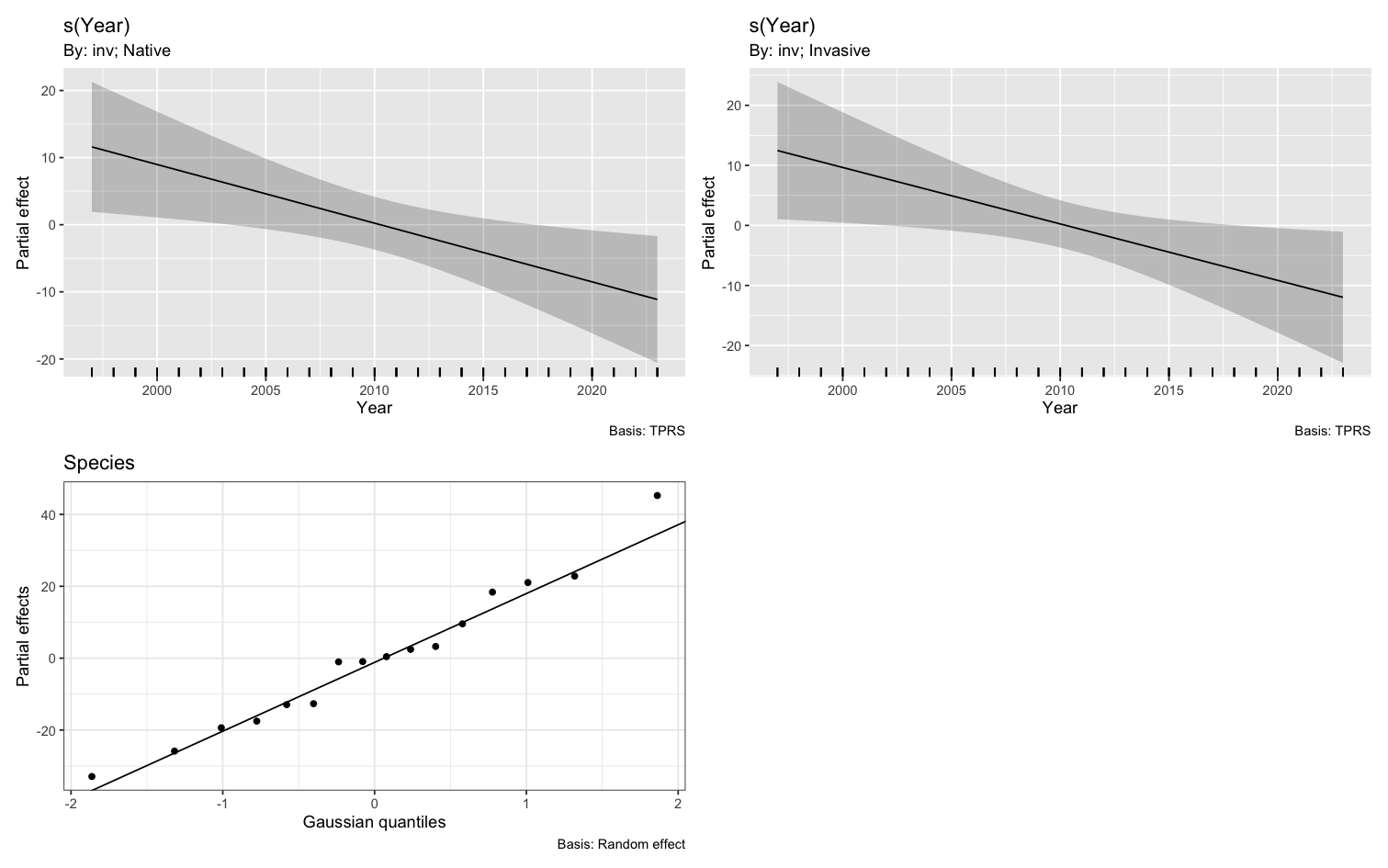


Supplemental 2. Estimated smooth effects from the generalized additive model showing temporal trends for the peak arrival for native and non-native fish species (i.e., the Julian date where 50% of cumulative individuals arrived). Top panels display the smooth effect of year on last occurrence by invasion status (Native, Non-native), with shaded areas representing 95% confidence intervals, and bottom shows the random effect estimates for individual species.


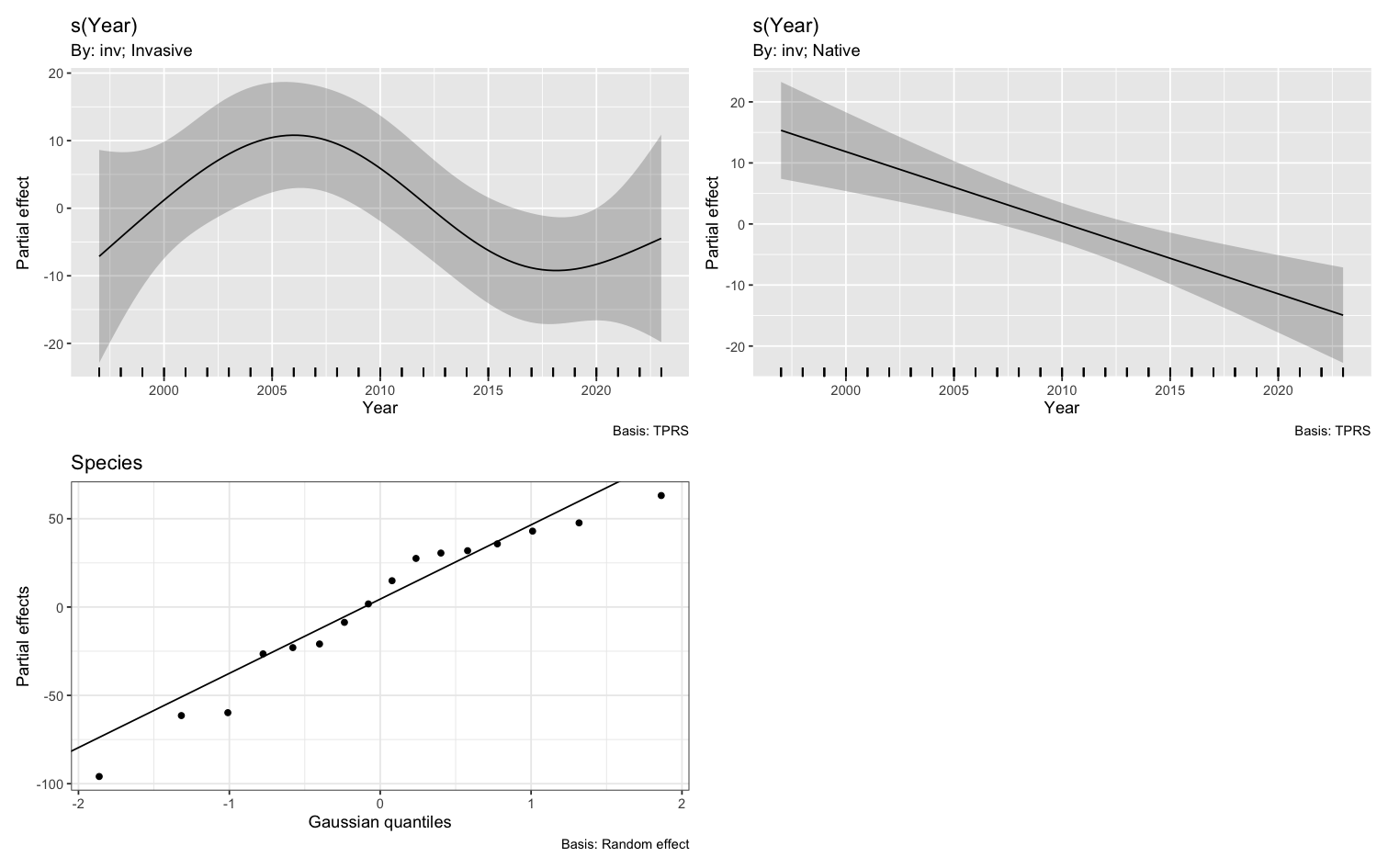


Supplemental 3. Estimated smooth effects from the generalized additive model showing temporal trends for the last arrival for native and non-native fish species. Top panels display the smooth effect of year on last occurrence by invasion status (Native, Non-native), with shaded areas representing 95% confidence intervals, and bottom shows the random effect estimates for individual species.


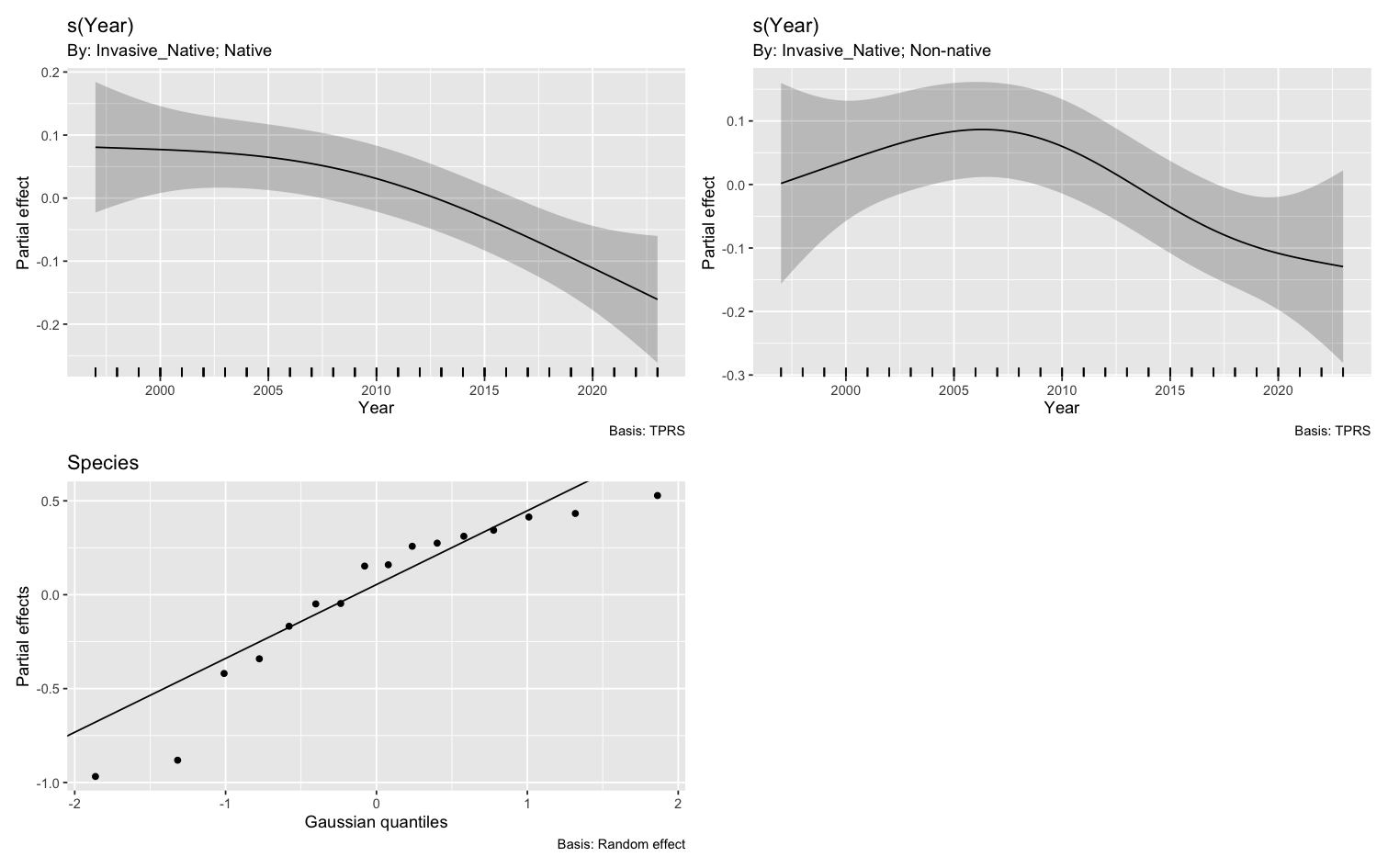


Supplemental 4. Estimated smooth effects from the generalized additive model showing temporal trends in the duration for native and non-native fish species. Top panels display the smooth effect of year on last occurrence by invasion status (Native, Non-native), with shaded areas representing 95% confidence intervals, and bottom shows the random effect estimates for individual species.
