# Supplementary material for: Climate Change Impacts on the Phenology of Laurentian Great Lakes Fishes
Source: Glob Chang Biol. 2025 Aug 19;31(8):e70436. doi: 10.1111/gcb.70436 (PMC12365581; doi:10.1111/gcb.70436)
Supplement: Supplementary file 1 — Data S1: gcb70436‐sup‐0001‐DataS1.zip. [file GCB-31-e70436-s001.zip › Supinfo/SummaryFile.pdf]

Column Name	Description
`Month`	Numeric representation of the month of observation (e.g., 7 = July)
`Year`	Year of data collection (e.g., 1986)
`date`	Full date of observation in `YYYY-MM-DD` format
`Temp`	Water temperature in degrees Celsius
`Conductivity`	Electrical conductivity of the water in microsiemens per centimeter (μS/cm)
`DO`	Dissolved oxygen concentration in milligrams per liter (mg/L)
