# Supplementary figures and images for: Climate Change Impacts on the Phenology of Laurentian Great Lakes Fishes
Source: Glob Chang Biol. 2025 Aug 19;31(8):e70436. doi: 10.1111/gcb.70436 (PMC12365581; doi:10.1111/gcb.70436)

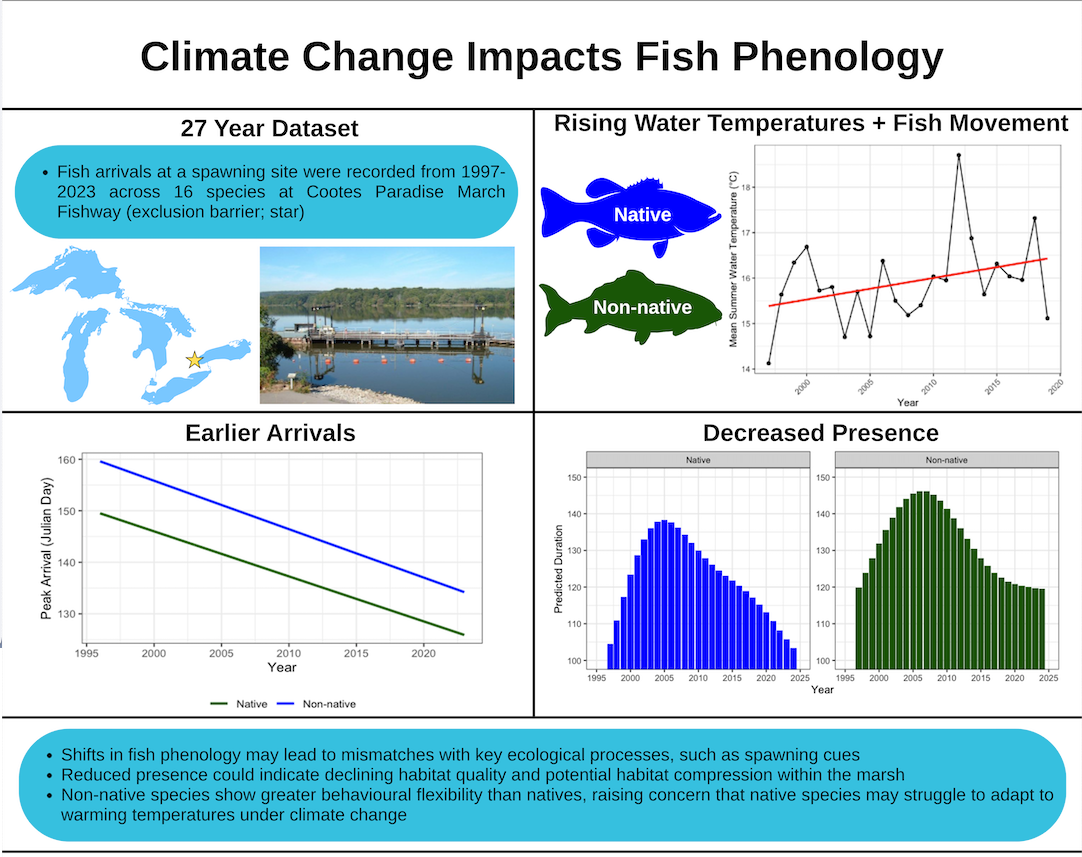

Supplement: Supplementary file 1 — Data S1: gcb70436‐sup‐0001‐DataS1.zip. [file GCB-31-e70436-s001.zip › Supinfo/Screenshot 2025-07-29 at 10.45.05 AM.png]

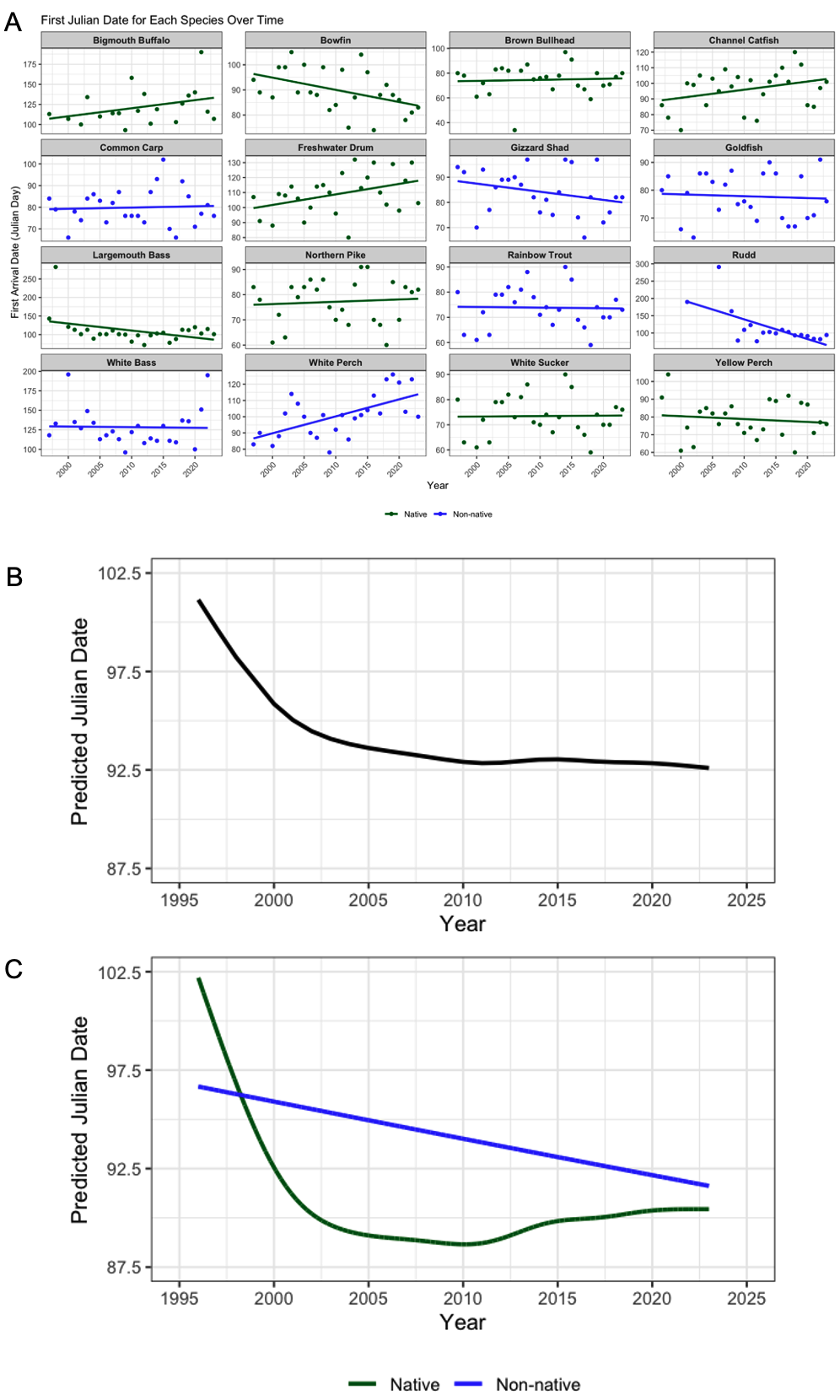

Supplement: Supplementary file 1 — Data S1: gcb70436‐sup‐0001‐DataS1.zip. [file GCB-31-e70436-s001.zip › Supinfo/Screenshot 2025-08-05 at 9.57.32 AM.png]

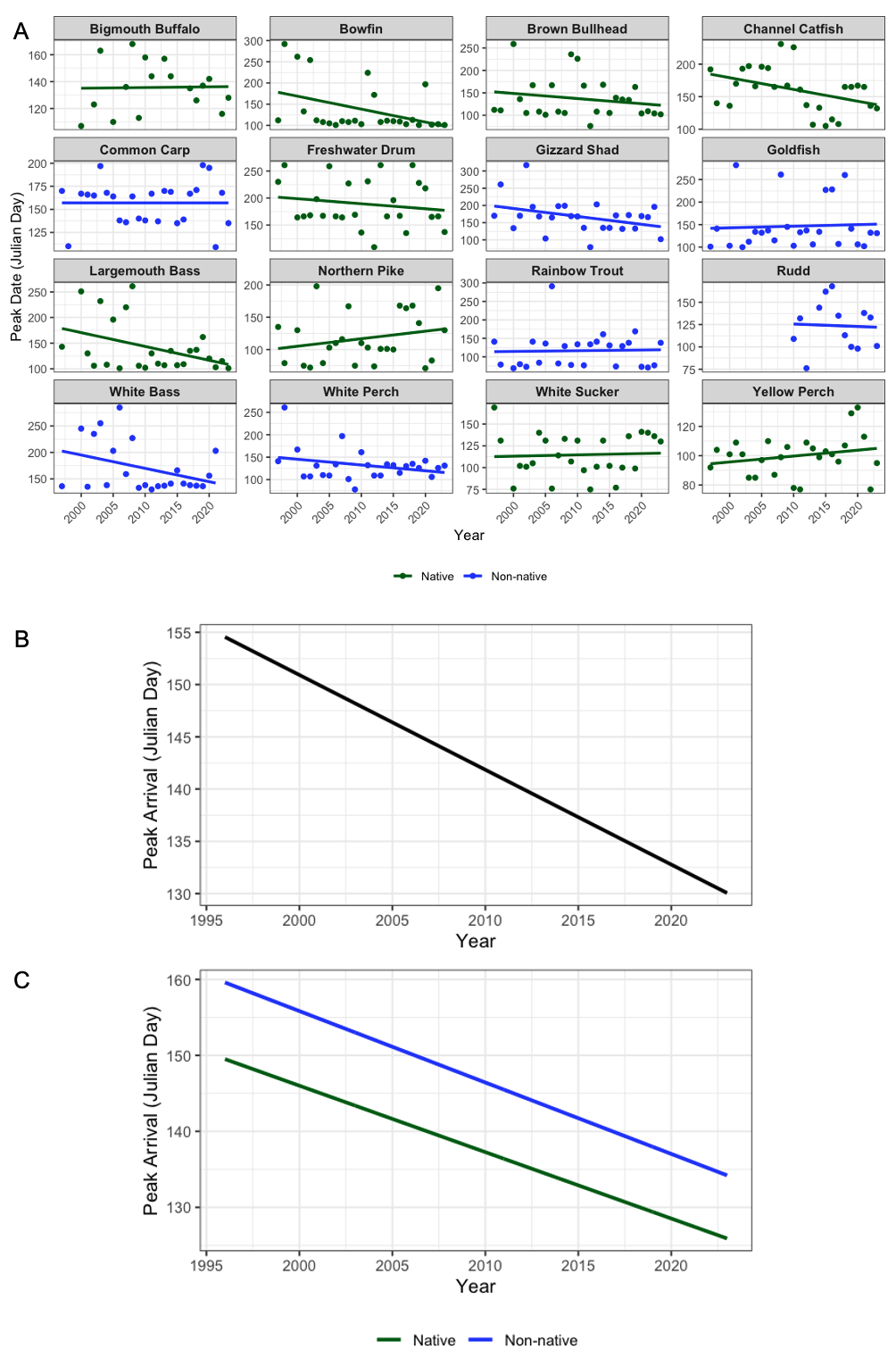

Supplement: Supplementary file 1 — Data S1: gcb70436‐sup‐0001‐DataS1.zip. [file GCB-31-e70436-s001.zip › Supinfo/Screenshot 2025-08-05 at 9.58.20 AM.png]

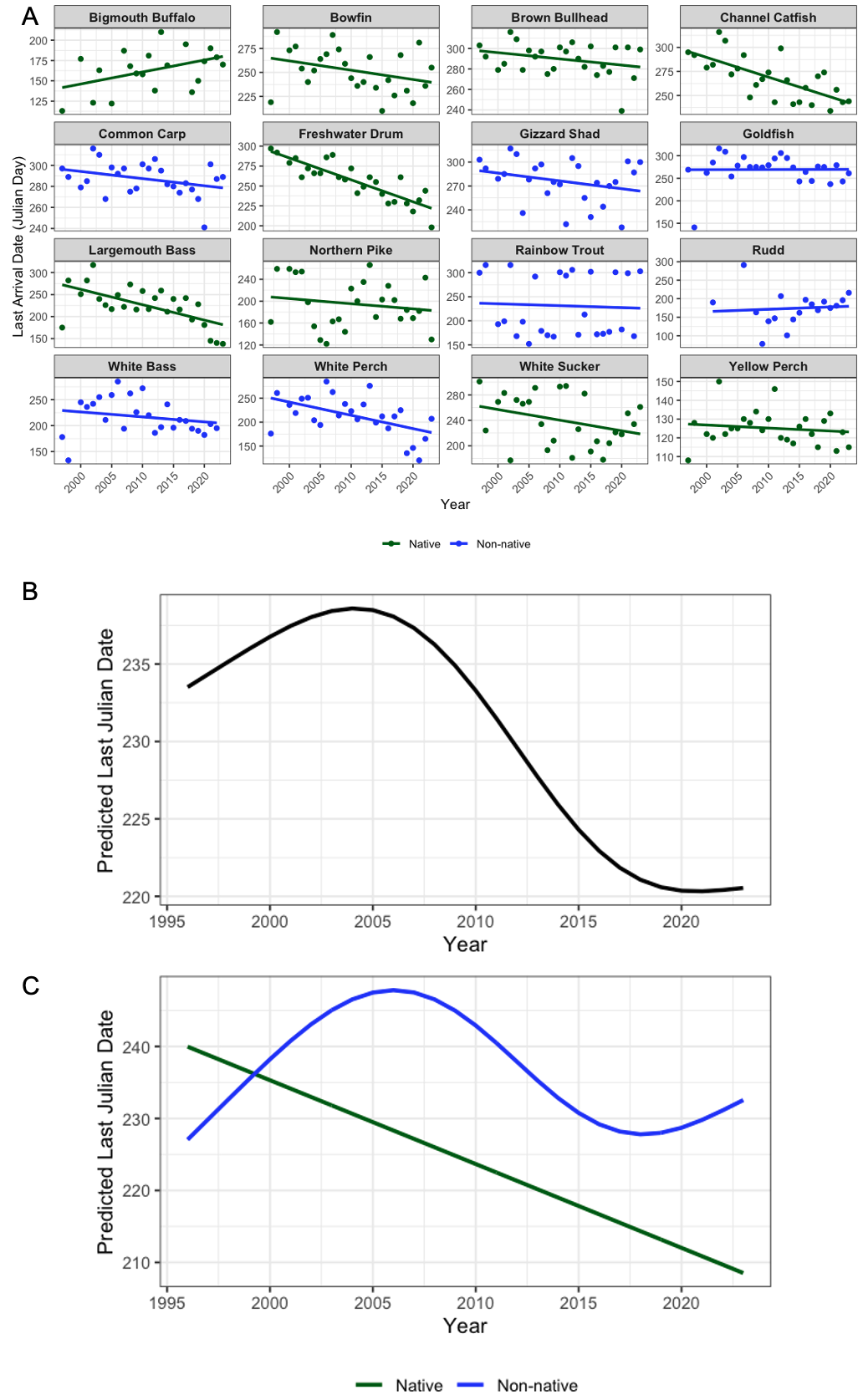

Supplement: Supplementary file 1 — Data S1: gcb70436‐sup‐0001‐DataS1.zip. [file GCB-31-e70436-s001.zip › Supinfo/Screenshot 2025-08-05 at 9.59.20 AM.png]
